# Effectiveness of elastic band training and group-based dance on physical-functional performance in older women with sarcopenia: a pilot study

**DOI:** 10.1186/s12889-023-17014-7

**Published:** 2023-10-27

**Authors:** Pablo Valdés-Badilla, Eduardo Guzmán-Muñoz, Jordan Hernandez-Martinez, Cristian Núñez-Espinosa, Pedro Delgado-Floody, Tomás Herrera-Valenzuela, Braulio Henrique Magnani Branco, José Zapata-Bastias, Hadi Nobari

**Affiliations:** 1 Department of Physical Activity Sciences, Faculty of Education Sciences, Universidad Católica del Maule, Talca, 3530000 Chile; 2 Sports Coach Career, School of Education, Universidad Viña del Mar, 2520000 Viña del Mar, Chile; 3 School of Kinesiology, Faculty of Health, Universidad Santo Tomás, Talca, 3460000 Chile; 4 School of Kinesiology, Faculty of Health Sciences, Universidad Autónoma de Chile, Talca, 3460000 Chile; 5 Programa de Investigación en Deporte, Sociedad y Buen Vivir, Universidad de los Lagos, Osorno, 5290000 Chile; 6 Department of Physical Activity Sciences, Universidad de Los Lagos, Osorno, 5290000 Chile; 7 School of Medicine, University of Magallanes, Punta Arenas, 6200000 Chile; 8 Centro Asistencial Docente e Investigación, Universidad de Magallanes, Punta Arenas, 6200000 Chile; 9 Interuniversity Center for Healthy Aging, Punta Arenas, 6200000 Chile; 10 Department of Physical Education, Sport, and Recreation, Universidad de La Frontera, Temuco, 4811230 Chile; 11 Department Physical Education and Sports, Faculty of Sport Sciences, University of Granada, Granada, 18011 Spain; 12 Department of Physical Activity, Sports and Health Sciences, Faculty of Medical Sciences, Universidad de Santiago de Chile (USACH), Santiago, 8370003 Chile; 13 Postgraduate Program in Health Promotion, Cesumar University, Maringá, 87050-390 Paraná Brazil; 14 Department of Exercise Physiology, Faculty of Educational Sciences and Psychology, University of Mohaghegh Ardabili, Ardabil, Iran; 15 Faculty of Sport Sciences, University of Extremadura, Cáceres, Spain

**Keywords:** Physical activity, Resistance training, Dance therapy, Older adults, Aging, Muscular atrophy

## Abstract

**Background:**

Sarcopenia is a syndrome associated with aging that causes progressive loss of skeletal muscle mass and muscle function. In this pilot study, we compared the effectiveness of elastic band training regarding group-based dance on fat mass, fat-free mass, handgrip strength (HGS; dominant and non-dominant hand), leg strength, timed up-and-go (TUG) and walking speed in older women with sarcopenia.

**Methods:**

This is a randomized controlled trial, single-blind, repeated measures of parallel groups (elastic band group: EBG, n = 21; group-based dance: GBD, n = 19), and a quantitative methodology. Three 60-minute sessions per week for 12 weeks were dedicated to the interventions with pre- and post-assessments. A two-factor mixed analysis of variance (ANOVA) model with repeated measures was performed to measure the group×time effect.

**Results:**

A significant interaction revealed for fat-free mass (F_1,16_= 18.91; *p* < 0.001; EBG + 10.9% vs. GBD − 1.97%), HGS dominant hand (F_1,16_= 7.44; *p* = 0.014; EBG + 10.9% vs. GBD + 0.59%), HGS non-dominant hand (F_1,16_= 6.41; *p* = 0.022; EBG + 10.21% vs. GBD + 3.80%), leg strength (F_1,16_= 17.98; *p* < 0.001; EBG + 9.1% vs. GBD + 3.83%), TUG (F_1,16_= 7.52; *p* = 0.014; EBG − 14.7% vs. GBD − 1.0%) and walking speed (F_1,16_ = 6.40; *p* = 0.019; EBG − 7.6% vs. GBD − 4.35%) in favor of EBG.

**Conclusion:**

Elastic band training produces significantly greater responses on physical-functional performance regarding group-based dance in older women with sarcopenia. On the other hand, the EBG revealed a significant improvement in fat-free mass and upper and lower limb muscle strength, as well as a significant decrease time in TUG, and walking speed. Elastic band exercise is a safe, easy, affordable, and effective physical activity strategy, according to the findings.

## Background

Sarcopenia is a syndrome leading to a gradual decline in skeletal muscle mass and function [1–4]. It is one of the leading causes of disability in older people and a sign of frailty [1]. Its prevalence depends on different factors such as sex, age, health status, lifestyle, pathologies (i.e., chronic diseases such as hypertension, diabetes, dyslipidemia), COVID-19, among others [3], which estimate its presence between 10 and 27% of the population over 60 years [4]. At the same time, the diversity of assessment and classification methods can underestimate or overestimate their presence [3]. This is of concern because sarcopenia can limit functioning in activities of daily living, affecting health-related quality of life in older people [5].

Among the main consequences of sarcopenia at the physical-functional level, a 47.3% lower handgrip strength (HGS) has been reported in older people using Brazil’s primary healthcare system [6]. While older people with sarcopenia from Japan and Korea demonstrate significantly less than normal lower limbs muscle strength [7] and lower performance in dynamic balance [8] and walking speed [9]. In addition, sarcopenia is associated with an increased risk of falls in older people [10]. In patients with sarcopenia, annual expenses can rise to roughly US$13,000 [11] due to factors that collectively affect functional independence [12] and raise the likelihood of hospitalization [13]. As a result, developing sarcopenia prevention and treatment strategies can increase older people’s physical fitness and lower healthcare expenses [11–13]. This knowledge has been verified across several epochs and cultural currents [14].

Contrarily, it has been shown that regular physical activity can decrease the negative effects of sarcopenia. For example, a systematic review with meta-analysis in sarcopenic older people reported that resistance training and concurrent training (i.e., resistance training combined with other exercises such as balance, muscular endurance, and aerobic training) significantly improved lower limbs muscle strength and gait speed compared to inactive control groups [13]. Another meta-analysis detect a large effect size on physical performance next to a moderate effect size on muscle strength in sarcopenic older people participating in various physical exercise programs [1]. While Ferreira, Scariot [15] found substantial improvements in HGS, skeletal muscle mass, and walking speed in sarcopenic older people who engaged in physical activity (both resistance training and non-resistance training) compared to control groups who did not. In older people with sarcopenia, several physical activity therapies result in desirable changes to body composition and physical fitness [1, 13, 15]. However, there have been no reported differences between multicomponent training and resistance training in healthy older people [16].

Following the studies mentioned above and systematic reviews, the international physical activity recommendations for older people include at least two weekly resistance training sessions encompassing large muscle groups [17, 18]. Governmental programs in Chile that promote physical activity for older women typically provide group-based dance and multicomponent training as practice options [19]. After 16 weeks of participation in governmental physical activity workshops, physical activity strategies (group-based dance and multicomponent training) have shown a significant reduction in anthropometric parameters such as body mass, waist circumference, waist-hip ratio and body mass index, as well as a significant improvement in tests related to muscle strength and flexibility of the lower and upper limbs and a reduction of time in the timed up-and-go test (TUG) in older people [19]. For their part, other interventions with Chilean older people have successfully used elastic bands in resistance training and multicomponent training, achieving significant improvements in HGS [20], lower limb muscle strength [21, 22], and a substantial reduction in fat mass [21]. While a recent meta-analysis by Sooktho, Songserm [23] showed that group-based dance programs are popular, safe and effective in promoting health status and improve muscle strength, balance and flexibility in older people, so far, it is unknown whether body composition and physical fitness in older women can be improved similarly with elastic band training program and group-based dance.

In light of this, the current pilot study compared the effectiveness of elastic band training regarding group-based dance on fat mass, fat-free mass, HGS (dominant and non-dominant hand), leg strength, TUG, and walking speed in older women with sarcopenia. Considering previous studies [24, 25], we hypothesis that, as compared to a group-based dance, the usage of elastic bands during exercise training is expected to provide noticeably greater gains in fat-free mass, HGS (in both dominant and non-dominant hands), and leg strength. However, the values obtained for fat mass, TUG, and walking speed are expected to be similar between the two groups. These outcomes could potentially strengthen the argument for incorporating resistance training in combating the adverse effects of sarcopenia.

## Methods

### Study design

This study is a randomized controlled trial, single-blind, repeated measures of parallel groups (elastic bands group: EBG; and group-based dance: GBD), and a quantitative methodology. The randomization was made using the randomizer internet site (https://www.randomizer.org). The methodology followed was the Consolidated Standards of Reporting Trials Statement (CONSORT) guidelines [26]. In addition, it has been registered in the Clinical Trial Protocol Registry and Results System (ClinicalTrials.gov) of the United States of America (code: NCT05275140; https://clinicaltrials.gov/search?cond=NCT05275140, first posted on March 11, 2022). The interventions occurred for 12 weeks, with three sessions a week (on Mondays, Wednesdays, and Fridays) of 60 min each. The evaluations were body composition (% to fat mass and fat-free mass), HGS in the dominant hand (kg) and non-dominant hand (kg), leg strength (kg), TUG (s), and walking speed (s). All assessments were conducted in the morning (between 9:00 to 11:30 h) and in the exact location (Laboratory, with the control of variables, temperature, and investigators that applied the devices in pre- and post-assessments). The older women did not present pain before the assessments or during the training sessions, without presenting musculoskeletal and/or cardiorespiratory injuries during the intervention.

### Participants

Forty-four older women initially participated in the intervention. The sample size calculation indicates the ideal number of participants per group (n *=* 12 older women). According to a prior study [21], for this calculation, an average difference of 0.55 s in the TUG test was used as the minimum difference required for substantial clinical relevance, with a standard deviation of 0.37 s, considering an alpha level of 0.05 with 95% power and an expected loss of 20%. GPower software (Version 3.1.9.6, Franz Faul, Universiät Kiel, Kiel, Germany) was used to calculate statistical power.

The inclusion criteria were: *(i)* older women aged between 60 and 90 years old; *(ii)* healthy by self-report (i.e., completion of the revised physical activity readiness questionnaire for older people); *(iii)* functionally independent, that is, have a score equal to or greater than 43 points in the Preventive Medicine Exam for the Older People (in Spanish, EMPAM) of the Ministry of Health of Chile [27]; *(iv)* older women with sarcopenia, which was identified to have two of the following three criteria: low muscle strength, low muscle quantity or quality, and low physical performance; according to the criteria of the established by Cruz-Jentoft [28], i.e., low muscle strength (HGS < 16 kg; chair stand test > 15 s for five rises), low muscle quantity or quality (appendicular skeletal muscle mass < 15 kg), and low physical performance (gait speed ≤ 0.8 m/s; and short physical performance battery ≤ 8 point score). The exclusion criteria following: *(i)* participants who presented any cardiovascular or respiratory pathology or musculoskeletal injury that prevented them from practicing physical activity; *(ii)* those who are permanently or temporarily unable to engage in physical activity; and *(iii)* those who presented moderate or severe cognitive impairment (≥ 15) assessed by the abbreviated Mini-Mental State Examination [29].

All participants had to accept the criteria for using and handling the data by signing an informed consent form authorizing the use of the information for scientific purposes. The research protocol was approved by the Research of Universidad Católica del Maule, Chile (approval number: N°29-2022) and developed following the Declaration of Helsinki with human beings.

### Primary outcomes

#### Body composition

Tetrapolar bioimpedance was used with eight tactile point electrodes on the InBody 570®, a body composition analyzer from Seoul, Korea, to determine the percentages of fat mass and fat-free mass. Every measurement was carried out in accordance with the International Society for Advances in Kinanthropometry (ISAK) guidelines [30].

#### Handgrip strength (HGS)

A portable dynamometer, manufactured by Patterson Medical, Sammons Preston Rolyan, Chicago, Illinois, USA, was used. The test was performed in a seated position, with the spine aligned, the shoulder in neutral position, the elbow flexed at 90 degrees to the side of the body, the forearm in neutral position and the wrist in neutral position, allowing the above suggestions [31]. According to the size of the hand, the position of the dynamometer was chosen to allow a secure grip of the instrument while maintaining adequate closure of the metacarpal phalangeal and interphalangeal joints, favoring contact between the first phalanx of the index finger and the thumb. Each person performed three rounds for each hand, with a 120-second rest between rounds.

#### Leg strength

A 5-minute overall warm-up was completed, followed by dynamic stretching for the lower limbs at low to moderate intensity (2 to 3 points in a rating of perceived exertion-RPE), followed by 90° leg flexions and extensions at a moderate intensity which was measured using the ten-point RPE [32]. The older women then sat on a leg press machine with the test load securely positioned in the starting position at moderate intensity (3 points of RPE). Following a previous study [33], the participants rated their feet on the leg press platform with their heels shoulder-width apart and below their knees. They were instructed to lift the weight off the rack, release the safety catch and prepare for the downward phase of the movement. The participant lowered the load to his buttocks until his knees were just below 90 degrees before concentrically contracting the leg muscles and extending the load back up, performing ten submaximal repetitions, after which he placed the weight securely on the rack before stepping off the machine. We continued with the same procedure but moved to a moderate-to-vigorous intensity load (3 to 5 points of RPE), executing five submaximal repetitions. In the end, we gave a rest of 2 to 3 min. We continued with a vigorous to very vigorous intensity load (7 to 9 points of RPE), performing two submaximal repetitions at the end, of which a rest of 3 to 5 min was granted. Finally, a repetition was performed with a maximum intensity load (10 points of RPE) which was recorded for statistical analysis.

#### Timed up-and-go (TUG)

This test involved rising from a chair with an armrest that was 50 cm from the ground, walking three meters, turning, and then returning to the starting position. The test was conducted in accordance with advice from Podsiadol and Richardson [34]. On a wooden indoor track, participants completed three trials with three minutes of rest in between. The best result was picked for statistical analysis after the time was estimated to within 0.01 s using single-beam infrared photoelectric cells (Brower Timing System, Salt Lake City, UT, USA).

#### Walking speed

The participants were told to walk for four meters twice with their all-out effort as quickly as they could without sprinting. Single-beam infrared photoelectric cells (Brower Timing System, Salt Lake City, UT, USA) were used to measure the time to the nearest 0.01 s. Starting from a standing position, each participant put their favorite foot forward and slightly back of the starting line. When participants voluntarily started the trial, which triggered timing, the test began. The timing gates were placed at the start of the race (0.3 m in front of the starting line) and at 4 m, about 0.7 m above the ground (i.e., hip level). The system used in this study was designed to accurately record trunk movements while minimizing the probability of spurious stimuli caused by limb movements. During the experimentation, participants concluded multiple trials with a 3-min rest period between each attempt. All tests were performed on an indoor wooden track. For data analysis, the best outcome from each participant was selected, agreeing with a previous research [35].

### Secondary outcomes

#### Anthropometric parameters

Bipedal height (cm) was measured with a stadiometer (Seca model 220, SECA, Hamburg, Germany; accuracy to 0.1 cm) and body mass (kg) was determined by donning the bare minimum of clothing and using a mechanical scale (Scale-tronix, Chicago, IL, USA; accuracy to 0.1 kg). Every measurement was carried out in accordance with the ISAK guidelines [30].

### Sociodemographic assessments

Age (years), academic level (primary, secondary, bachelor, master, Ph.D.), and civil status (married, separated, widowed, single, others).

### Interventions

**Fig. 1:**
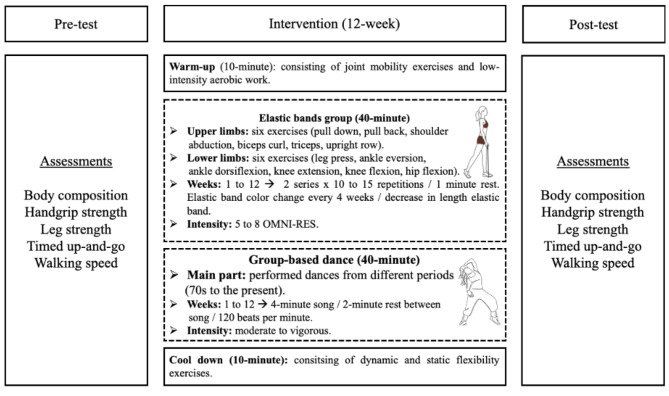
Assessments and progression for interventions. Legends: OMNI-RES: OMNI-Resistance Exercise Scale of perceived exertion

The participants’ vital signs were measured using an automated pressure monitor (08 A, CONTEC, Alsdorf, Germany) to determine their systolic and diastolic blood pressure as well as their resting heart rate prior to each session of elastic band training and group-based dance. The elastic bands training and group-based dance had a general structure that started with a 10-minute warm-up that included joint mobility exercises and a low-intensity aerobic program; after that, the central part (elastic bands and group-based dance) was developed for 40 min, and it was designed to end with a 10-minute cool-down that included dynamic and static flexibility exercises. Figure 1 provides a summary of the measures and intervention progression.

Elastic band training is based on previous researches [21, 36] that demonstrated that it is safe and effective for older people. Using the Thera Band® rubber band system (Hygenic Corporation, Akron, Ohio). The colors of the elastic bands (yellow, red, green, blue, black, silver, and gold), each corresponding to a certain range of endurance strength, were used to indicate training loads. The OMNI-Resistance Exercise Scale of perceived exertion was used to control the resistance training intensity, which ranged from moderate to vigorous (5–8 points) [37]. Six upper limbs strength exercises (pulldown, pullback, shoulder abduction, biceps curl, triceps, upright) and 6 for lower limbs (leg press, ankle eversion, ankle dorsiflexion, knee extension, knee flexion, and hip flexion). The older women started with the lowest resistance (yellow color), achieving a 10-repetition maximum (10 RM) of an upper and/or lower limbs exercise, proceeding to the next elastic band color until the 10 RM could not be produced. The final elastic band was the one chosen to begin the training program. During each training session, the older women reacted to two sets at an intensity equivalent to 100% (10 RM) with a 1-minute rest between each exercise. For 12 weeks of intervention, the volume was constant using two sets of 10 to 15 repetitions for each upper and lower limbs exercises. The maximum strength (with 10 RM) was measured with an elastic band of higher resistance. If they could achieve the 10 RM, they proceeded in color every four weeks; if they could not progress to a band of higher resistance, the length of the band was shortened by half. In this way, it remained until 12 weeks of intervention.

The group-based dance consisted of a warm-up using dances from the 1960 and 1970 s at low to moderate intensities (10 min). The central part (40 min) consisted of dances of moderate to high intensity from different periods (the 70s to the present), where each song lasted approximately 4 min with 2-minute rest between each piece. To finish (10 min), cool down through relaxing music and execute dynamic and static flexibility exercises. The intensity remained moderate to vigorous, having a heart rate < 120 beats per minute [19].

### Statistical analysis

Firstly, the Shapiro-Wilk test was used to determine the data normality. After the normality confirmation, the values were reported as mean and standard deviation. A two-factor repeated measures of analysis of variance (ANOVA) was used to measure the group×time effect of all variables. When the group×time interaction was significant, the Bonferroni post-hoc test was performed to establish intragroup differences (pre vs. post), while intergroup differences (EBG vs. GBD) were determined with the Mann-Whitney U test. The effect size (ES) was determined through Cohen’s *d*, considering a small (0.20–0.49), moderate (0.50–0.79), or large (> 0.80) effect [38]. A significant difference was established for all analyses at 5%. Data were analyzed with SPSS 25.0 statistical software (SPSS 25.0 for Windows, SPSS Inc., Chicago, IL, USA).

## Results

**Fig. 2:**
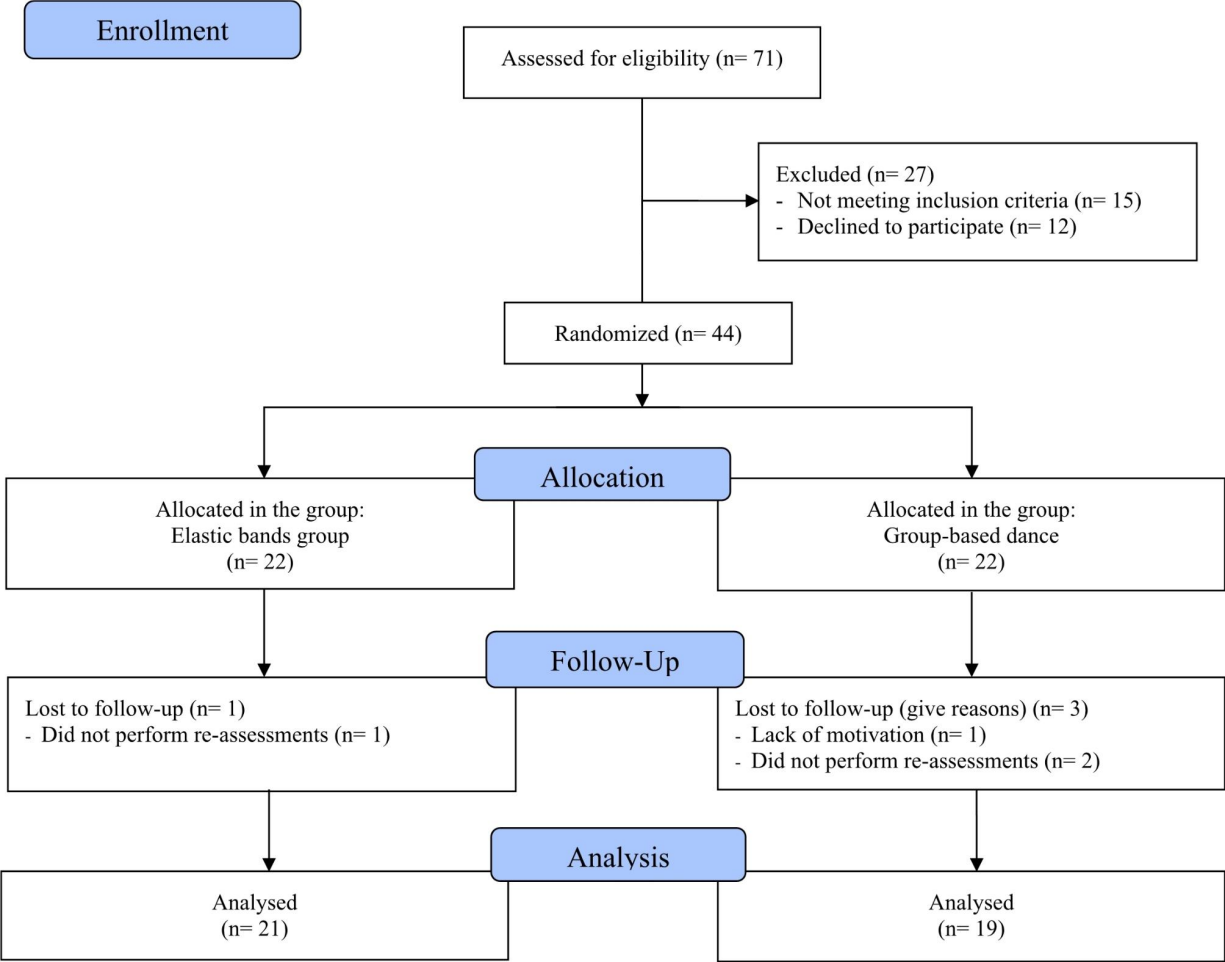
Study flowchart, based on CONSORT

As depicted in Fig. 2, to be included in the final analyses, participants who met the inclusion criteria must also complete ≥ 85% of all training sessions, i.e., ≥ 30 of 36 sessions, and attend all assessment sessions. Of the 44 older women primarily considered for inclusion in the study, one was excluded due to lack of motivation, and three did not perform the re-assessments. Hence, 21 older women were analyzed in the EBG and 19 in the GBD.

The baseline secondary outcomes revealed that, in general, the older women with sarcopenia analyzed in this study had a mean age of 73.26 ± 8.35 years. Furthermore, 44% of them had a primary academic level, 46% had a secondary academic level, and 10% had a bachelor’s degree. Additionally, it can be noted that 68% were married, 24% were separated, and 8% were widowed (Table 1).

**Table 1:** Baseline anthropometric parameters and sociodemographic assessments of older women with sarcopenia

Variable	Assessment	EBG (n = 21)	GBD (n = 19)	General sample (n = 40)
Age (years)	Mean (SD)	73.91 (8.27)	72.85 (8.67)	73.26 (8.35)
Antrophometric parameters	Bipedal height (cm)	153.96 (7.72)	150.00 (10.63)	152.16 (9.39)
	Body mass (kg)	72.42 (15.63)	69.36 (13.41)	70.97 (14.72)
Academic level	Primary (%)	23	21	44
	Secondary (%)	26	20	46
	Bachelor (%)	4	6	10
	Postgraduate (%)	0	0	0
Civil status	Married (%)	38	30	68
	Separated (%)	13	11	24
	Widowed (%)	6	2	8
	Single (%)	0	0	0
	Others (%)	0	0	0

EBG: elastic bands group. GBD: group-based dance. SD: standard deviation

The group×time repeated measures ANOVA revealed a significant interaction for fat-free mass (F_1,16_= 18.91; *p* < 0.001), HGS dominant hand (F_1,16_= 7.44; *p* = 0.014), HGS non-dominant hand (F_1,16_= 6.41; *p* = 0.022), leg strength (F_1,16_= 17.98; *p* < 0.001), TUG (F_1,16_= 7.52; *p* = 0.014) and walking speed (F_1,16_= 6.40; *p* = 0.019) in favor of EBG. The pre- and post-intervention analyses (Bonferroni post hoc test) showed that in the EBG, there were significant improvements in the assessments of fat-free mass, HGS, leg strength, TUG, and walking speed. After 12 weeks of training with elastic bands, an increase of 10.9% in fat-free mass (*p* < 0.001; ES = 0.68), 10.9% in HGS dominant hand (*p* = 0.003; ES = 0.53), 10.21% in HGS non-dominant hand (*p* = 0.008; ES = 0.53), and 9.1% of leg strength (*p* < 0.001; ES = 0.35). On the other hand, the performance in the TUG tests and walking speed showed significant improvements in the performance of the EBG, reflected in the 14.7% decrease in the TUG (*p* < 0.001; ES = 0.58) and 7.6% in walking speed (*p* = 0.037; ES = 0.34). For the GBD, no significant differences were observed in any of the assessments carried out (Table 2).

**Table 2:** Intragroup post hoc analyses in body composition and physical fitness in older women with sarcopenia

Assessment	Group	PRE		POST		*p* Value	Change (%)	ES
		Mean	SD	Mean	SD			
Fat-free mass (%)	EBG (n = 21)	22.10	3.43	24.50	3.67	< 0.001^***^	10.86	0.68
	GBD (n = 19)	21.28	2.88	20.86	2.82	0.999	-1.97	0.15
Fat mass (%)	EBG (n = 21)	31.79	12.09	31.20	11.22	0.999	-1.86	0.05
	GBD (n = 19)	27.39	10.36	28.11	10.95	0.999	2.63	0.07
HGS Dominant hand (kg)	EBG (n = 21)	16.27	3.43	18.05	3.24	0.003^**^	10.94	0.53
	GBD (n = 19)	17.01	3.54	16.91	2.86	0.999	0.59	0.03
HGS Non-dominant hand (kg)	EBG (n = 21)	15.18	3.09	16.73	2.73	0.008^**^	10.21	0.53
	GBD (n = 19)	14.75	1.98	15.31	3.53	0.999	3.80	0.19
Leg strength (kg)	EBG (n = 21)	42.52	12.01	46.39	9.69	< 0.001^***^	9.10	0.35
	GBD (n = 19)	45.65	12.18	47.40	11.74	0.473	3.83	0.15
TUG (s)	EBG (n = 21)	10.36	3.04	8.84	2.08	< 0.001^***^	-14.67	0.58
	GBD (n = 19)	10.04	2.72	9.94	2.50	0.762	-1.00	0.04
Walking speed (s)	EBG (n = 21)	2.75	0.69	2.54	0.53	0.037^*^	-7.64	0.34
	GBD (n = 19)	2.99	0.72	2.86	0.54	0.863	-4.35	0.20

SD: standard deviation. EBG: elastic bands group. GBD: group-based dance. ES: effect size. HGS: handgrip strength. TUG: timed up-and-go. *p < 0.05. ** p < 0.01. ***p < 0.001. Bonferroni post hoc test.

**Fig. 3:**
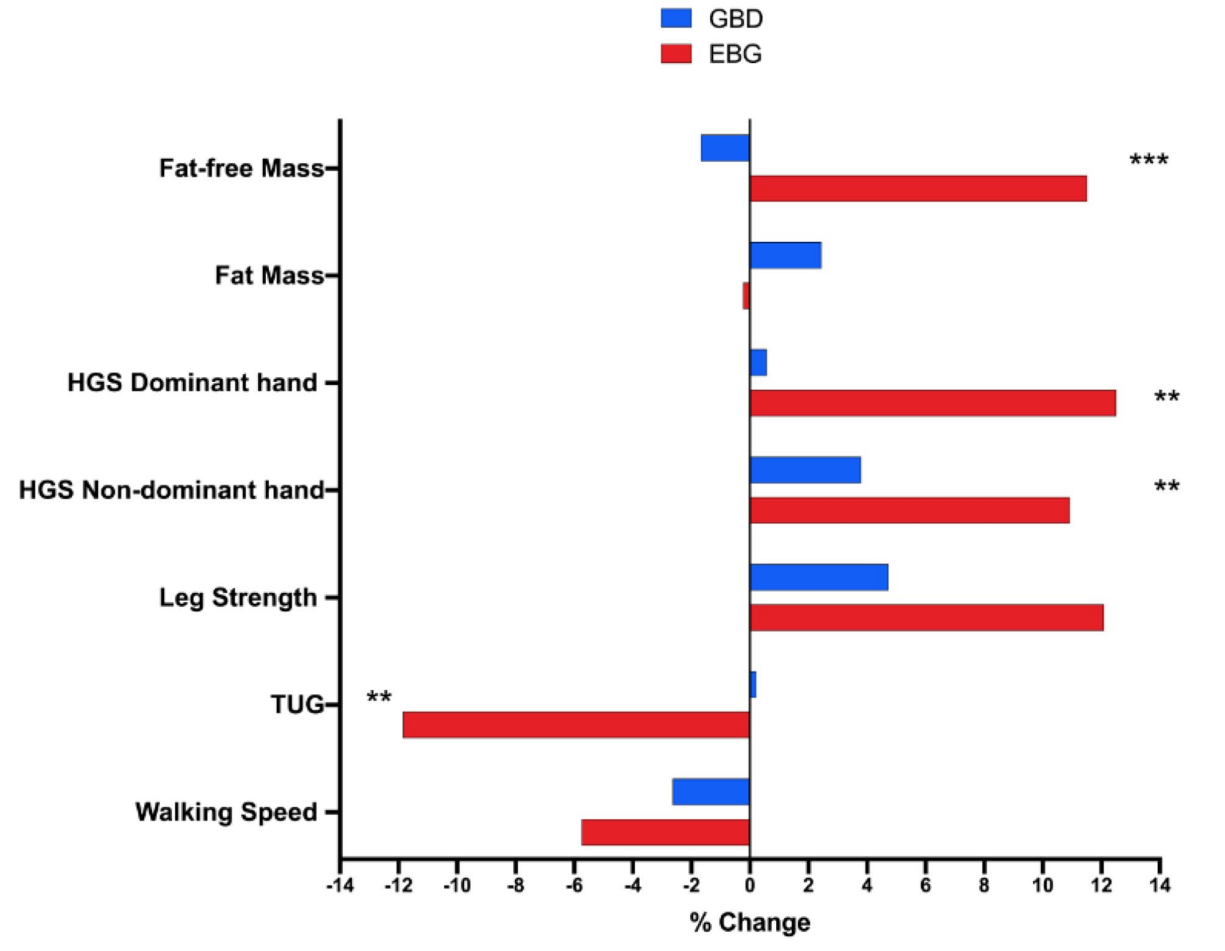
Differences between elastic bands training regarding to group-based dance on physical-functional performance in older women with sarcopenia. Legends: EBG: elastic bands group. GBD: group-based dance. HGS: handgrip strength. TUG: timed up-and-go. **p < 0.01. ***p < 0.001. Mann-Whitney U test

The comparison between groups (EBG vs. GBD) is displayed in Fig. 3, in which significant differences (Mann-Whitney U test) are shown in favor of EBG in the fat-free mass (*p* < 0.001; ES = 1.29), HGS dominant hand (*p* = 0.006; ES = 0.83), HGS non-dominant hand (*p* = 0.009; ES = 0.53), and TUG (*p* = 0.008; ES = 0.83). For the assessments of fat mass (*p* = 0.441; ES = 0.24), leg strength (*p* = 0.072; ES = 0.57), and walking speed (*p* = 0.439; ES = 0.24) no significant differences.

## Discussion

The main aim of this pilot study was to assess the effectiveness of elastic band training regarding group-based dance on fat mass, fat-free mass, HGS, leg strength, TUG, and walking speed in older women with sarcopenia. The main findings show that adding elastic bands to a physical training regimen enhances fat-free mass, HGS (both dominant and non-dominant hands), leg strength, TUG, and walking speed. Similar outcomes have previously been noted in older people, where six weeks of elastic bands exercise led to statistically significant improvements in TUG and fat mass [21]. Similarly, another study demonstrated that six months of elastic bands training improved quadriceps and hamstring strength, upper limbs muscle strength, cardiorespiratory fitness, walking speed, and general physical fitness [39].

The strength of the muscles in the upper and lower limbs significantly enhanced, according to the findings of our study. The upper limb values grew by roughly 10.5%, whereas the lower limbs values rose by about 9%. However, the GBD’s muscle strength did not increase. These results are in line with those of Chen, Wu [40], who demonstrated that using an elastic band elevated HGS in pre-fragile older people by 19% during the first four weeks of intervention and by 30% after the first eight. The older women who were tested had a baseline sarcopenia status, which may have contributed to the lower percentage increase in muscle strength seen [41, 42]. Elastic bands training offers a changing resistance stimulus throughout the range of motion of a particular exercise, in contrast to typical resistance training (gym equipment and dumbbells), where the external load is constant throughout the whole range of motion [43]. According to Bellar, Muller [44], the large variety of stimuli that causes elastic bands would promote the brain adaptations that enhance the various expressions of muscle strength. One study showed that elastic band exercises had greater shoulder muscle activation (lower trapezius, anterior deltoid, and middle deltoid) than dumbbell use, attributed to the neuromuscular stimulation produced by variable resistance loading [45]. In addition, it has been observed that training with elastic devices (bands or tubes) can improve the conduction velocity of motor units [46]. These improvements are possibly associated with greater recruitment of fast motor units in exercises with elastic bands. They could explain the increase in muscle strength observed in the older women evaluated in our study.

After six weeks of elastic bands training, older people with sarcopenia increased their fat-free mass by 11%. Although during resistance training, it has been seen that during the first weeks, the neural adaptations are greater than those of hypertrophy [47], in our study, it was possible to observe that the increase in fat-free mass was significant for older women with sarcopenia who underwent the intervention. After 12 weeks of elastic band exercise, sarcopenic older people were also shown to have increased fat-free mass in a related study [48]. Elastic band training did not, however, result in a substantial improvement in fat-free mass in healthy older people [21]. Given that the stimulation can cause early changes in fat-free mass, reducing the risk of falls and favoring physical-functional performance and health-related quality of life in this population, this situation highlights the value of strength training in older people with sarcopenia [49].

Regarding TUG and walking speed tests, a significant decrease was observed in the time it took older women with sarcopenia who were trained with elastic bands to perform the tests. The unstable modality of the elastic bands produces greater muscle activation than traditional training, allowing a more efficient motor performance during walking and maintaining postural balance [21]. This aspect coincides with the improvement in motor functionality obtained in other studies of older people [21, 48, 50]. Also, increased muscle strength has been related to improved postural balance [51]. Therefore, the greater muscle strength exhibited by, the older women in this study could explain the changes in TUG and walking speed (dynamic balance). Changes in balance ability in response to increases in strength exhibited by older women are possibly due to the further development of torque rates caused by the increased cross-sectional area of type II fibers [52]. Favorable facts because dynamic balance is a complex activity that involves large muscle groups, coordination, and sensory function [53] and is related to lower risk of fall in older people [54].

On the other hand, the duration of the elastic band and group-based dance interventions used in our study—12 weeks, three sessions per week lasting 60 min each—are in line with the guidelines for physical activity for older people [17, 18]. All of the sessions planned for our intervention were overseen and guided by professionals in physical activity. Relevant fact, according to Lacroix, Hortobagyi [55], supervised physical activity interventions (balance/resistance training) have been shown to produce significantly better results than unsupervised interventions in the areas of static steady-state balance, dynamic steady-state balance, proactive balance, and muscle strength/power. In this context, it has been suggested that to maximize gains in strength and muscle morphology from resistance training programs targeted at older people, it is crucial to take the dose-response relationship into account [56].

This study’s potential limitations include a lack of control over food intake and a failure to complete a food record (to understand the participants’ dietary profiles, including their protein, carbohydrate, lipid, and micronutrient intake), both of which could affect the participants’ body composition and levels of physical fitness. The lack of inclusion of male participants did not bring the possibility to expand the range of these findings and allow for a more comprehensive comparison between different groups and sex; in addition to considering only the older women who completed ≥ 85% of all training sessions, which could limit the analysis. Therefore, to study potential differences in the efficacy of elastic band training on enhancing physical-functional performance and preventing sarcopenia between sexes, future research may benefit from using a broad sample population that includes both men and women. The inclusion of an active control group and participant randomization, which improves the study’s internal consistency, are its key advantages. The external validity is also increased through the use of protocolized, validated assessments and scientifically based training design.

## Conclusion

In comparison to group-based dance, elastic band training produces significantly greater responses in fat-free mass, HGS, and a significant decrease in time of TUG in older women with sarcopenia. On the other hand, the EBG revealed a significant decrease in fat-free mass, time in TUG, and walking speed, as well as a significant improvement in upper and lower limbs muscle strength. Elastic band exercise is a safe, easy, affordable, and effective physical activity strategy, according to the findings.

## Data Availability

The datasets generated during and/or analyzed during the current research are available from the Corresponding author upon reasonable request.

## Abbreviations

HGS: Handgrip strength
TUG: Timed up-and-go
EBG: Elastic band group
GBD: Group-based dance
ISAK: International Society for Advances in Kinanthropometry
RPE: Rating of perceived exertion
OMNI-RES: OMNI-Resistance Exercise Scale of perceived exertion
10 RM: 10-repetition maximum

## References

[1] Escriche-Escuder A, Fuentes-Abolafio IJ, Roldan-Jimenez C, Cuesta-Vargas AI (2021). Effects of exercise on muscle mass, strength, and physical performance in older adults with Sarcopenia: a systematic review and meta-analysis according to the EWGSOP criteria. Exp Gerontol.

[2] Billot M, Calvani R, Urtamo A, Sánchez-Sánchez JL, Ciccolari-Micaldi C, Chang M (2020). Preserving mobility in older adults with physical frailty and sarcopenia: opportunities, challenges, and recommendations for physical activity interventions. Clin Interv Aging.

[3] Papadopoulou SK, Sarcopenia (2020). A contemporary health problem among older adult populations. Nutrients.

[4] Petermann-Rocha F, Balntzi V, Gray SR, Lara J, Ho FK, Pell JP (2022). Global prevalence of Sarcopenia and severe Sarcopenia: a systematic review and meta‐analysis. J cachexia Sarcopenia Muscle.

[5] Valdés-Badilla P, Alarcón-Rivera M, Hernandez-Martinez J, Herrera-Valenzuela T, Branco BHM, Núñez-Espinosa C (2022). Factors associated with poor health-related quality of life in physically active older people. Int J Environ Res Public Health.

[6] de Carli Tonial P, Colussi EL, Sant’Anna Alves AL, Stürmer J, Bettinelli LA (2020). Prevalencia De La Sarcopenia entre Los ancianos usuarios del sistema de atención primaria. Nutrición Hospitalaria.

[7] Kato T, Ikezoe T, Tabara Y, Matsuda F, Tsuboyama T, Ichihashi N (2022). Differences in lower limb muscle strength and balance ability between sarcopenia stages depend on sex in community-dwelling older adults. Aging Clin Exp Res.

[8] Shin HE, Kim M, Won CW (2022). Differences in characteristics between older adults meeting criteria for Sarcopenia and possible Sarcopenia: from research to primary care. Int J Environ Res Public Health.

[9] Kitamura A, Seino S, Abe T, Nofuji Y, Yokoyama Y, Amano H (2021). Sarcopenia: prevalence, associated factors, and the risk of mortality and disability in Japanese older adults. J cachexia Sarcopenia Muscle.

[10] Yeung SS, Reijnierse EM, Pham VK, Trappenburg MC, Lim WK, Meskers CG (2019). Sarcopenia and its association with falls and fractures in older adults: a systematic review and meta-analysis. J cachexia Sarcopenia Muscle.

[11] Gani F, Buettner S, Margonis GA, Sasaki K, Wagner D, Kim Y (2016). Sarcopenia predicts costs among patients undergoing major abdominal operations. Surgery.

[12] Cebrià i Iranzo MA, Arnal-Gómez A, Tortosa-Chuliá MA, Balasch-Bernat M, Forcano S, Sentandreu-Mañó T (2020). Functional and clinical characteristics for predicting Sarcopenia in institutionalised older adults: identifying tools for clinical screening. Int J Environ Res Public Health.

[13] Lu L, Mao L, Feng Y, Ainsworth BE, Liu Y, Chen N (2021). Effects of different exercise training modes on muscle strength and physical performance in older people with Sarcopenia: a systematic review and meta-analysis. BMC Geriatr.

[14] Shams A, Nobari H, Afonso J, Abbasi H, Mainer-Pardos E, Pérez-Gómez J (2021). Effect of aerobic-based exercise on psychological well-being and quality of life among older people: a Middle East study. Front Public Health.

[15] Ferreira LF, Scariot EL, da Rosa LHT. The Effect of different Exercise Programs on Sarcopenia Criteria in Older people: a systematic review of systematic reviews with Meta-analysis. Arch Gerontol Geriatr. 2022:104868. 10.1016/j.archger.2022.104868 36402001

[16] Lemos ECWM, Guadagnin EC, Mota CB. Influence of strength training and multicomponent training on the functionality of older adults: systematic review and meta-analysis. Volume 22. Revista Brasileira de Cineantropometria & Desempenho Humano; 2020.

[17] Fragala MS, Cadore EL, Dorgo S, Izquierdo M, Kraemer WJ, Peterson MD et al. Resistance training for older adults: position statement from the national strength and conditioning association. J Strength Conditioning Res. 2019;33(8). 10.1519/JSC.0000000000003230 31343601

[18] Bull FC, Al-Ansari SS, Biddle S, Borodulin K, Buman MP, Cardon G (2020). World Health Organization 2020 guidelines on physical activity and sedentary behaviour. Br J Sports Med.

[19] Valdés-Badilla P, Guzmán-Muñoz E, Ramírez-Campillo R, Godoy-Cumillaf A, Concha-Cisternas Y, Ortega-Spuler J (2020). Changes in anthropometric parameters and physical fitness in older adults after participating in a 16-week physical activity program. Revista De La Facultad De Medicina.

[20] Hernandez-Martinez J, Castillo-Cerda M, Vera-Assaoka T, Carter-Thuillier B, Herrera-Valenzuela T, Guzmán-Muñoz E (2022). Warm-Up and handgrip strength in physically inactive Chilean older females according to Baseline Nutritional Status. Int J Environ Res Public Health.

[21] Miranda-Aguilar D, Valdés-Badilla P, Herrera-Valenzuela T, Guzmán-Muñoz E, Magnani Branco B, Méndez-Rebolledo G et al. ¿ Bandas elásticas o Equipos De Gimnasio Para El entrenamiento de adultos mayores? Retos: nuevas tendencias en educación física, deporte y recreación. 2020(37):370–8.

[22] Vargas-Vitoria R, Larena JA, Rodríguez M, Arellano R, Valdés-Badilla P. Efectos De Un programa multicomponente sobre medidas antropométricas, condición física y calidad de vida relacionada con la salud en personas mayores. Nutrición Clínica Y Dietética Hospitalaria. 2021;41(1).

[23] Sooktho S, Songserm N, Woradet S. Suksatan WJAogm, research. A Meta-analysis of the effects of Dance Programs on Physical Performance: Appropriate Health Promotion for healthy older adults. 2022;26(3):196. 10.4235/agmr.22.0066 PMC9535373 36064303

[24] Barajas-Galindo DE, Arnáiz EG, Vicente PF, Ballesteros-Pomar MD. Effects of physical exercise in Sarcopenia. A systematic review. Endocrinología, Diabetes Y Nutrición (English ed). 2021;68(3):159–69. 10.1016/j.endien.2020.02.007 34167695

[25] Chen N, He X, Feng Y, Ainsworth BE, Liu Y (2021). Effects of resistance training in healthy older people with Sarcopenia: a systematic review and meta-analysis of randomized controlled trials. Eur Rev Aging Phys Activity.

[26] Turner L, Shamseer L, Altman DG, Weeks L, Peters J, Kober T et al. Consolidated standards of reporting trials (CONSORT) and the completeness of reporting of randomised controlled trials (RCTs) published in medical journals. Cochrane Database of Systematic Reviews. 2012(11). 10.1002/14651858.MR000030.pub2 PMC7386818 23152285

[27] Ministerio de Salud. Manual de Aplicación del Examen de Medicina Preventiva del Adulto Mayor. In: Subsecretaria de salud pública, editor. https://www.minsal.cl.2013.

[28] Cruz-Jentoft AJ, Bahat G, Bauer J, Boirie Y, Bruyère O, Cederholm T, Cooper C, Landi F, Rolland Y, Sayer AA, Schneider SM, Sieber CC, Topinkova E, Vandewoude M, Visser M, Zamboni M (2019). & writing Group for the European Working Group on Sarcopenia in Older people 2 (EWGSOP2), and the Extended Group for EWGSOP2. Sarcopenia: revised European consensus on definition and diagnosis. Age Ageing.

[29] Jiménez D, Lavados M, Rojas P, Henríquez C, Silva F, Guillón M (2017). Evaluación Del minimental abreviado de la evaluación funcional del adulto mayor (EFAM) como screening para la detección de demencia en la atención primaria. Revista médica De Chile.

[30] Marfell-Jones MJ, Stewart A, de Ridder J. International standards for anthropometric assessment2012.

[31] Fess E (1992). Grip Strength.

[32] Borg GA (1982). Psychophysical bases of perceived exertion. Med sci Sports Exerc.

[33] Parrino RL, Strand KL, Hockman AC, Signorile JF (2021). Leg press and chest press strength normative values by half-decades in older persons. Exp Gerontol.

[34] Podsiadol D, Richardson S (1991). The time up & go: a test of basic functional mobility for frail elderly persons. J Am Geriat Society.

[35] Lyons JG, Heeren T, Stuver SO, Fredman L (2015). Assessing the agreement between 3-meter and 6-meter walk tests in 136 community-dwelling older adults. J Aging Health.

[36] Egaña M, Reilly H, Green S (2010). Effect of elastic-band-based resistance training on leg blood flow in elderly women. Appl Physiol Nutr Metab.

[37] Colado JC, Pedrosa FM, Juesas A, Gargallo P, Carrasco JJ, Flandez J (2018). Concurrent validation of the OMNI-Resistance Exercise Scale of perceived exertion with elastic bands in the elderly. Exp Gerontol.

[38] Cohen J (1992). A power primer. Psychol Bull.

[39] Oesen S, Halper B, Hofmann M, Jandrasits W, Franzke B, Strasser E-M (2015). Effects of elastic band resistance training and nutritional supplementation on physical performance of institutionalised elderly—A randomized controlled trial. Exp Gerontol.

[40] Chen R, Wu Q, Wang D, Li Z, Liu H, Liu G et al. Effects of elastic band exercise on the frailty states in pre-frail elderly people. Physiother Theory Pract. 2019. 10.1080/09593985.2018.1548673 30741081

[41] Seo M-W, Jung S-W, Kim S-W, Jung HC, Kim D-Y, Song JKJIJER et al. Comparisons of muscle quality and muscle growth factor between sarcopenic and non-sarcopenic older women. 2020;17(18):6581. 10.3390/ijerph17186581 PMC7558172 32927586

[42] Buckinx F, Aubertin-Leheudre, MJIJoWsH. Sarcopenia in menopausal women: current perspectives. 2022:805 – 19. 10.2147/IJWH.S340537 PMC9235827 35769543

[43] Andersen V, Prieske O, Stien N, Cumming K, Solstad TEJ, Paulsen G et al. Comparing the effects of variable and traditional resistance training on maximal strength and muscle power in healthy adults: a systematic review and meta-analysis. J Sci Med Sport. 2022. 10.1016/j.jsams.2022.08.009 36130847

[44] Bellar DM, Muller MD, Barkley JE, Kim C-H, Ida K, Ryan EJ (2011). The effects of combined elastic-and free-weight tension vs. free-weight tension on one-repetition maximum strength in the bench press. J Strength Conditioning Res.

[45] Bergquist R, Iversen VM, Mork PJ, Fimland MS (2018). Muscle activity in upper-body single-joint resistance exercises with elastic resistance bands vs. free weights. J Hum Kinetics.

[46] Melchiorri G, Rainoldi A (2011). Muscle fatigue induced by two different resistances: Elastic tubing versus weight machines. J Electromyogr Kinesiol.

[47] Schoenfeld BJ (2010). The mechanisms of muscle hypertrophy and their application to resistance training. J Strength Conditioning Res.

[48] Liao C-D, Tsauo J-Y, Huang S-W, Ku J-W, Hsiao D-J, Liou T-H (2018). Effects of elastic band exercise on lean mass and physical capacity in older women with sarcopenic obesity: a randomized controlled trial. Sci Rep.

[49] Rodrigues F, Domingos C, Monteiro D, Morouço P (2022). A review on aging, Sarcopenia, falls, and resistance training in community-dwelling older adults. Int J Environ Res Public Health.

[50] Motalebi SA, Amirzadeh Iranagh J, Mohammadi F, Cheong LS (2018). Efficacy of elastic resistance training program for the institutionalized elderly. Top Geriatric Rehabilitation.

[51] Wang Q, Li L, Mao M, Sun W, Zhang C, Mao D (2022). The relationships of postural stability with muscle strength and proprioception are different among older adults over and under 75 years of age. J Exerc Sci Fit.

[52] Pavol MJ, Owings TM, Foley KT, Grabiner MD (2002). Influence of lower extremity strength of healthy older adults on the outcome of an induced trip. J Am Geriatr Soc.

[53] Padilla Colón CJ, Sánchez Collado P, Cuevas MJ (2014). Beneficios Del entrenamiento de fuerza para la prevención y tratamiento de la Sarcopenia. Nutrición Hospitalaria.

[54] Valdés-Badilla P, Gutiérrez-García C, Pérez-Gutiérrez M, Vargas-Vitoria R, López-Fuenzalida A (2019). Effects of physical activity governmental programs on health status in Independent older adults: a systematic review. J Aging Phys Act.

[55] Lacroix A, Hortobagyi T, Beurskens R, Granacher U (2017). Effects of supervised vs. unsupervised training programs on balance and muscle strength in older adults: a systematic review and meta-analysis. Sports Med.

[56] Borde R, Hortobágyi T, Granacher U (2015). Dose–response relationships of resistance training in healthy old adults: a systematic review and meta-analysis. Sports Med.

